# “Nudge” in the clinical consultation – an acceptable form of medical paternalism?

**DOI:** 10.1186/1472-6939-15-31

**Published:** 2014-04-17

**Authors:** Ajay Aggarwal, Joanna Davies, Richard Sullivan

**Affiliations:** 1 Department of Research Oncology, King’s Institute of Cancer Policy, Guys Hospital, 3rd Floor Bermondsey Wing, Great Maze Pond, London SE1 9RT, England; 2 London School of Economics and Political Science, Houghton Street, London WC2A 2AE, England; 3 Department of Palliative Care, Guys & St Thomas’ NHS Trust, Guys Hospital, Great Maze Pond, London SE1 9RT, England

**Keywords:** Nudge, Libertarian paternalism, Communication, Framing, Shared decision making, Medical consultation

## Abstract

**Background:**

Libertarian paternalism is a concept derived from cognitive psychology and behavioural science. It is behind policies that frame information in such a way as to encourage individuals to make choices which are in their best interests, while maintaining their freedom of choice. Clinicians may view their clinical consultations as far removed from the realms of cognitive psychology but on closer examination there are a number of striking similarities.

**Discussion:**

Evidence has shown that decision making is prone to bias and not necessarily rational or logical, particularly during ill health. Clinicians will usually have an opinion about what course of action represents the patient’s best interests and thus may “frame” information in a way which “nudges” patients into making choices which are considered likely to maximise their welfare. This may be viewed as interfering with patient autonomy and constitute medical paternalism and appear in direct opposition to the tenets of modern practice. However, we argue that clinicians have a responsibility to try and correct “reasoning failure” in patients. Some compromise between patient autonomy and medical paternalism is justified on these grounds and transparency of how these techniques may be used should be promoted.

**Summary:**

Overall the extremes of autonomy and paternalism are not compatible in a responsive, responsible and moral health care environment, and thus some compromise of these values is unavoidable. Nudge techniques are widely used in policy making and we demonstrate how they can be applied in shared medical decision making. Whether or not this is ethically sound is a matter of continued debate but health care professionals cannot avoid the fact they are likely to be using nudge within clinical consultations. Acknowledgment of this will lead to greater self-awareness, reflection and provide further avenues for debate on the art and science of clinical communication.

## Background

Helping individuals to make decisions which promote their own welfare, without limiting their freedom of choice is one of the hallmarks of “libertarian paternalism” (LP) or “asymmetric paternalism” [1,2].

Paternalism is often considered a serious threat to the autonomy and choice of an individual, and is associated with perceived authoritarian policies such as prohibition [3]. However LP aims to provide a framework where individuals make decisions which benefit themselves and society, whilst still maintaining a range of available options. In other words by changing the “choice architecture” for decision making, individuals can be “nudged” into making the right choices [4].

The premise for this approach is based on our understanding of behavioural economics [5] which has characterised decision making processes and the biases which may impact on rational decision leading to “reasoning failure” [6]. This can result in choices that negatively impact on welfare, [7] which is particularly relevant to medical practice where patients are often required to weigh up risks of survival, toxicity and quality of life when making treatment decisions. Under these circumstances patients may also be influenced by an array of emotions such as fear and grief [6].

Studies of cancer patients have found that demands for particular treatments do not come from a neutral evaluation of risks and benefits but rather from a perception of hope even when faced with a high likelihood of major toxicity and low benefit [8]. Further inconsistencies in decision making may arise from previous experiences, particular if these have been unpleasant [6]. Such findings are entirely in line with Prospect Theory where decisions made under conditions of gains and losses are very different, and not subject to a state of maximising personal utility [9].

During the 1960’s and 70’s there was a considerable backlash towards medical paternalism, with a move towards “whole person” medicine [10]. The onus shifted to the individual to take responsibility for decisions relating to their health needs. Cassileth [11] pithily summarised this new era, “The current approach derives from a new emphasis on individual responsibility and the right to control one’s own living and dying. Informed consent, open communication, full disclosure and patient information are expressions of the contemporary focus”.

Informed decision making requires the provision of comprehensive and objective information [12]. However patients may struggle with probabilities, over-estimating their level of risk of disease and the potential benefits of treatment [13]. It has been argued that too many options can have negative consequences resulting in individuals using heuristics (rules of thumb) to counter the numerous choices on offer, resulting in suboptimal decisions [14]. Individuals may suffer from “myopia” where they are not able to imagine decisions that will impact them in the future, specifically not anticipating how their preferences may change over time [15]. An extension of this phenomenon is known as “hyperbolic discounting” where one places a disproportionate weight on the present, relative to future costs and benefits [16]. An example would be an individual continuing with excessive or unhealthy eating habits given that the effects of obesity are delayed, but food consumption results in immediate gratification. People also prefer the “status quo”, in other words their current situation over an alternative, even if superior options exist [17]. This inertia effect has been termed the the default bias [17,18].

In clinical practice doctors appreciate these challenges and apply many of the principles of LP in the process of shared and informed decision making. Here we explore whether doctors are justified in “framing” the discussion and delivery of information to achieve what their professional judgement would consider the optimal choice for the patient, or is this indeed a case of paternalism and thus a threat to patient autonomy?

## Discussion

The following hypothetical case scenarios provide examples of the use of “nudge” in a clinical consultation and will be used to explore the issues involved.

### Consultation A

Eve is a 42 year old lady with a node positive, Grade III hormone resistant breast cancer that has been fully excised. The National Institute of Health and Care Excellence (NICE) would recommend that she proceed with adjuvant chemotherapy followed by radiotherapy [19]. She does not wish to have chemotherapy due to witnessing her mother’s bad experiences during chemotherapy for lung cancer. She is particularly concerned about hair loss, infection and the impact on her quality of life.

The doctor explains that the treatments for lung cancer and breast cancer do not utilise the same drugs and thus the side effects are different. Fatigue is expected but this may not impact on her day to day activities with some patients able to continue working whilst on treatment. The main reason for offering chemotherapy is to prevent recurrent disease, which if it did occur would be incurable. Her risk of relapse is 80% without treatment. Chemotherapy doubles her chance of being alive in 10 years time.

The aim would be to treat side effects such as nausea and vomiting with medication. She has an 85% likelihood of not having any serious infection [20]. The doctor explains that her risk of alopecia is high, but her hair is highly likely to grow back on completion of treatment. The patient explains that she is confused, and also upset given the memories of her mother’s suffering. The doctor advises the patient to avoid making any immediate decisions. He provides Eve with written information on chemotherapy and arranges a review in one week’s time.

### Consultation B

Jennifer is a 60 year old lady with a node negative, Grade I hormone sensitive, breast cancer that has been fully excised. NICE recommend treatment with adjuvant radiotherapy and anti-oestrogen hormonal treatment for 5 years [19]. However, Jennifer sees chemotherapy as an essential part of cancer treatment in order to give her the best chance of cure. She bases this opinion on her discussions with other patients who have had breast cancer, all of whom had chemotherapy which they tolerated well.

The doctor explains she has a very high chance of cure. The benefit from chemotherapy is minimal, specifically if 100 women with her cancer were treated with chemotherapy, 99 patients would receive no benefit. Information regarding the toxicity of treatment is provided which includes severe sickness (20%), infection requiring hospitalisation (15%), hair loss (85%) and fatigue which is experienced by the majority of patients [20]. The patient is still adamant she wishes to have chemotherapy. The doctor explains that her risks of complications outweigh the benefits and that she would be at undue risk of toxicity and in some cases death. It is recommended that she attend in one week’s time after reviewing the information provided.

### Consultation review

The scenarios described are two extremes but demonstrate medical consultations in which the pros and cons of treatment are discussed. Firstly one can see the impact of frame effects. This refers to the different methods by which logically equivalent information can be conveyed or presented and has been shown to influence decision making [21]. The reference point of Eve in scenario A is her prior negative experience of chemotherapy after witnessing her mother’s treatment. An attempt to shift her from this occurred when the differences between the two diseases and their treatments were highlighted. The toxicity data was positively framed by emphasising that the risk of not developing a serious infection was 85%. Although fatigue may be an issue, attempts were made to change her reference point to the “norm” by explaining that some patients are able to work during treatment, which is likely to alter her interpretation of the debilitating effect of chemotherapy.

By comparison, negative framing was used to explain the rates of toxicity including alopecia and infection in scenario B. It was also used to convey the message about the poor efficacy of chemotherapy with 99/100 having no benefit compared to 1/100. Describing negatively framed risk information numerically has a greater impact on treatment choices than a verbal risk description [22]. In case A, her risk of sickness was described as high and in scenario B, a figure of 20% was used to convey the same information.

Prospect Theory suggests that highlighting perceived losses associated with inaction are more likely to motivate action in risky situations than perceived gains [6,9]. In Scenario A, it was explained that inaction (i.e. a choice of no chemotherapy) would result in an 80% risk of relapse. Loss framing has been shown to have a greater impact than positive framing, particular when looking at screening uptake [23]. In scenario A, the method of presenting the additional benefit of chemotherapy was presented as a relative risk which has been shown in trials to have a greater impact than presentation of absolute risks [24].

Another nudge strategy in these vignettes involved the regulation of timing decisions [25]. Both patients were asked to consider their options in light of the new information provided during the consultation. This allows personal reflection and also a resetting of their frame of reference which may be influenced by prior negative or positive experiences. This “cooling off” period is therefore designed to optimise patient choice [4].

### Analysis

Critics of nudge policies suggest that they do not unbias individual’s decision making, in other words correct the causes of reasoning failure, but rather utilise these biases to trick them into a certain decision [26]. They contend that the use of such mechanisms impacts on an individual’s autonomy as they are not fully in control of their actions, and there should be greater transparency [27]. Freedom of choice is a core tenet of the LP philosophy however there remains incongruity between the “nominal freedom of choice” and the “effective freedom of choice.” For example auto-enrolled opt out schemes (e.g. organ donation) result in only a small proportion of people leaving the scheme, due to exploitation of their status quo bias [28].

It may be that using paternalism and autonomy as the two overriding, principles is overly simplistic. It has been suggested that it is perhaps more accurate to consider nudge in terms of “manipulation,” [29] which describes someone influencing a situation in order to achieve a desired outcome, without making it apparent that this is occurring. Manipulation, does suggest that the motivation is personal gain and the contrast here is that in these examples the manipulation of information is believed to be for the patient’s benefit. However, irrespective of motivation and outcome, the technique will be the same and this raises some interesting ethical issues and reminds us that while in health policy these techniques appear to have been used with good intentions there is nothing stopping the same techniques being used for bad ones.

An example would be subliminal messaging in advertising where the motivation is profit yet the method still relies on taking advantage of a predictable human behavioural response. Thaler and Sunstein defend equating nudge with manipulation by arguing that one difference between the two lies in the fact that nudge techniques should be transparent and publicly defensible [25]. If we apply this to the clinical consultations in our article the doctor is giving their opinion openly however is not necessarily being transparent about the way in which he is choosing to deliver information. The word manipulation itself is certainly more emotive and arguably has more negative connotations than paternalism, which implies protection and care, principles which sit more comfortably in the ethos of healthcare.

However, perhaps health care professionals are being paternalistic to each other by shying away from the concept of manipulation and hiding behind the more acceptable defence of paternalism rather than openly acknowledging the use of nudge in clinical encounters. An example might be if the clinician acknowledged to the patient that they were delivering the information in a specific way; for example by saying “I am giving you the information in this way to help you understand why I think this is the best course of action.” If the motivation is for the patients benefit and the clinician is open about the way he or she delivers information then claims of manipulation can be refuted and patient autonomy maximised.

This brings us to autonomy. If we were to look at autonomy in its purest form rationality does not come into it. Only the individual to whom that decision relates can truly appreciate their own values and preferences and thus the best interests that will be unique to them. Therefore any attempt to influence this will result in a decision that is not truly in their best interests. However being a patient with a potentially life threatening condition puts a considerable strain on their ability to process information. The reasons and evidence for this are discussed elsewhere in the paper and this raises the question whether it is the duty of a doctor to recognise and correct reasoning failure in their patients? If so would this be considered a paternalistic act?

Autonomy is central to patient care and a patient is entitled to make seemingly unwise decisions or refuse life sustaining treatment so long as they have the mental capacity to do so. For patients deemed not to have capacity to make specific decisions statute law allows health care professionals to make those decisions in the patient’s best interests. (Mental Capacity Act 2005) However, Komrad argues that illness represents a temporary loss of patient autonomy mediated by vulnerability, fear, loss of self esteem, and physical or mental incapacity. As a result to some degree paternalistic input is justified to restore an individual’s autonomy [30].

Medical training aims to equip clinicians with appropriate skills to deal holistically with the complexities of medical practice. Medical decision making should be a collaborative process between patient and health care professional. However, that clinicians should not have an opinion on what constitutes a preferred treatment option for their patients defies the objectives of their training and the development of clinical judgement. Of course patient choice is paramount, but it is important to note that to date medical professionals maintain the right not to give treatments that are not beneficial, or even harmful, even if this is perceived as directly interfering with patient autonomy, although clearly the aim should be to avoid situations with such conflicting opinions. This position has been upheld by case law (R on the application of Burke v The General Medical Council [2005] EWCA Civ 1003) and in the recent case of Mr James [31] and is detailed in the General Medical Council’s ethical guidance [32].

If we look again at scenario B; despite her requests, the administration of chemotherapy is likely to cause harm and even be potentially fatal without any clear benefit and seems to be unequivocally not to be in her best interests. The patient’s reasoning failure should not allow her to insist on a treatment which is frankly harmful even if this may be considered to be “overtly paternalistic.” Collaborative working is vital, but fundamentally there is a duty of care and responsibility to patients which cannot be absolved.

A potential criticism to this argument is that it is naive to think that it is only patients who are subject to external influences. Doctors themselves, despite their training, are human and are certainly not immune from biases and this has been well documented [33]. Clearly both patients and clinicians will be subject to any number of biases related to personal factors. However in addition to these, patients may be expected to encounter more predictable biases related to their adverse health status. These are more relevant to the concept of nudge as they allow some prediction of a typical behavioural response in a given situation.

To promote informed decision making, autonomy and effective collaboration, information sharing is crucial [34]. However, the question arises as to whether all patients strive for full information and control over choice, or whether some prefer guidance. Is the role of the doctor just to provide information? The evidence suggests that a patient’s desire for information acts as a proxy for building a trusting relationship with their doctor [35]. There is little evidence that the information, although valued, informs actual choices [36]. Other patients do not necessarily wish to have information and complain of overload, and the notion that all information is universally positive has been challenged [37]. For example patients with prostate cancer describe regrets and isolation when deciding on the appropriate course of curative treatment, given there is not a clear superiority between options. However, patients are still encouraged to take responsibility for their treatment choice in a field where optimum management is unclear [38].

The value of shared decision making models has also been questioned [39]. Specifically the model defines both parties as equals, however both physicians and patients have defined their relationship as “asymmetric” [40]. Other elements of a doctor-patient relationship are hope and care [41], which are intuitive responses by the health professional to the needs of the patient. This in itself represents a desire from both patients and doctors for the patient to be offered some degree of protection [42].

## Summary

Cognitive psychology and behavioural science have provided a framework for understanding key processes in patient decision making. Nudge techniques are widely used in policy making and we have demonstrated how they can be applied in shared medical decision making. Whilst this is not taught within the medical curriculum, clinicians have developed techniques for countering reasoning failure in the clinical setting. Whether these are classified as overt “nudge techniques” or soft paternalism the aim is for the doctor and patient to work in close collaboration to achieve decisions that are in the patient’s best interests. Collaboration should allow preservation of freedom of choice but at the same time not result in the professional forgoing their clinical responsibility or professional judgement. Overall the extremes of autonomy and paternalism are not compatible in a responsive, responsible and moral health care environment, and thus some compromise of these values is unavoidable. Whether or not using nudge is ethically sound is a matter of opinion but health care professionals cannot shy away from the fact they are likely to be using it within clinical practice. Acknowledging this will hopefully lead to greater transparency and self awareness and provide further avenues for debate on the art and science of clinical communication.

## Competing interests

The authors declare that they have no competing interest.

## Authors’ contributions

AA and JD were involved in the conception and design of the manuscript. AA performed the initial literature review and drafted the initial manuscript. JD and RS revised the manuscript critically. All authors read and approved the final manuscript.

## Authors’ information

AA is a Specialist Registrar in Clinical Oncology undertaking an MSc in Health, Population and Society at the London School of Economics and Political Science. JD is a Specialist Registrar in Palliative Care and has a MA in Medical ethics and law. RS is the director of the King’s Health Partners Institute of Cancer Policy, and Professor of Cancer Policy & Global Health.

## Pre-publication history

The pre-publication history for this paper can be accessed here:

http://www.biomedcentral.com/1472-6939/15/31/prepub
